# Acylglycerol Kinase-Targeted Therapies in Oncology

**DOI:** 10.3389/fcell.2021.659158

**Published:** 2021-07-22

**Authors:** Binxiang Chu, Zhenghua Hong, Xiaohe Zheng

**Affiliations:** ^1^Department of Orthopedic, Taizhou Hospital of Zhejiang Province Affiliated to Wenzhou Medical University, Linhai, China; ^2^Department of Pathology, Taizhou Hospital of Zhejiang Province Affiliated to Wenzhou Medical University, Linhai, China

**Keywords:** acylglycerol kinase, mitochondria, Sengers syndrome, oncogenesis, metabolism

## Abstract

Acylglycerol kinase (AGK) is a recently discovered mitochondrial lipid kinase, and mutation of its gene is the fundamental cause of Sengers syndrome. AGK is not only involved in the stability of lipid metabolism but also closely related to mitochondrial protein transport, glycolysis, and thrombocytopoiesis. Evidence indicates that AGK is an important factor in the occurrence and development of tumors. Specifically, AGK has been identified as an oncogene that partakes in the regulation of tumor cell growth, invasion, metastasis, and drug resistance. The versatility of AGK and its unique role in different types of cancerous and normal cells greatly piqued our interest. We believe that AGK is a promising target for cancer therapy. Therefore, this review summarizes the main research advances concerning AGK, including the discovery of its physiological/pathogenic mechanisms, and provides a reference for the feasible evaluation of AGK as a therapeutic target for human diseases, particularly tumors.

## Introduction

**FIGURE 1:**
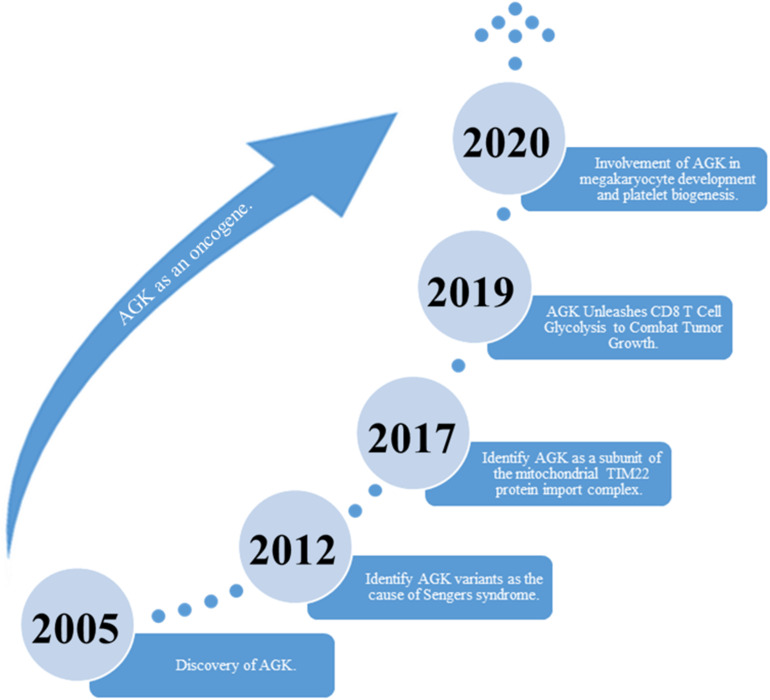
The discovery of AGK’s historical breakthrough.

Acylglycerol kinase (AGK), also named multi-substrate lipid kinases (MULK), was initially found through its mutation in Sengers syndrome (Mayr et al., 2012). In the past 10 years, new insights into the biological function of AGK and its significant role in human diseases (Abu El-Asrar et al., 2012, 2013, 2014) (especially tumors) have been established (Figure 1). AGK is renowned as a mitochondrial lipid kinase (Bektas et al., 2005). However, in-depth studies have proclaimed that AGK has a variety of kinase-independent biological responses in multitudinous cells, including as a subunit of the mitochondrial translocase of the inner membrane 22 (TIM22) complex (Kang et al., 2017; Vukotic et al., 2017), which involves transmembrane proteins [such as mitochondrial carrier family members (SLC25A family)] entering the mitochondrial interior from the cytoplasm (Kang et al., 2018; Jackson et al., 2021). Also, AGK partakes in glycolysis in CD8 T cells (Hu et al., 2019) and thrombocytopoiesis (Jiang et al., 2020). Importantly, AGK has been identified as a key oncogene that is highly expressed in a range of tumor types, such as prostate cancer (Spiegel and Milstien, 2005; Nouh et al., 2009; Zeng et al., 2009), breast cancer (Wang et al., 2014), cervical squamous cell carcinoma (Sun et al., 2016), and esophageal squamous cell carcinoma (ESCC) (Chen et al., 2013). AGK participates in the regulation of multiple signaling pathways during tumor occurrence and progression, which makes it a potential target for tumor therapy. In this paper, we comprehensively summarize the structure, function, and regulatory mechanism of AGK, focusing on its regulatory role in the tumor signaling pathway. Also, we describe the latest research results from AGK studies and prospects for future challenges.

## AGK Gene, Protein Structure, and Function

### Gene

The human AGK gene is located on chromosome 7q34 and contains 15 exons and 1,269 bases, making it highly similar (90% similarity) to AGK in mice. The AGK gene in mice is located on chromosome 6, comprising 16 exons and 1,263 bases in an open reading frame.

### Protein Structure

**FIGURE 2:**
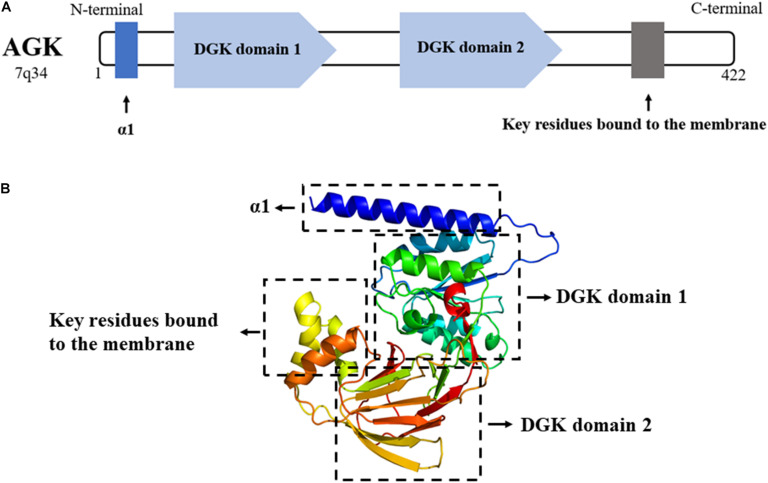
**(A)** AGK is composed of 422 amino acids and contains an α1 helix, a DGK domain 1, a DGK domain 2, and a C-terminal key residue bound to the membrane. DGK, diacylglycerol kinase. **(B)** 3D structure of AGK generated by X-ray crystal diffraction and visualized in Phyre2 (Kelley et al., 2015).

AGK is a nuclear gene-encoded protein mainly located in the mitochondrial membrane, while very recent studies suggest that a portion of AGK is localized elsewhere in the cell (Hu et al., 2019; Jiang et al., 2020). As a newly discovered member of the lipid kinase family, AGK is diffusely expressed in the heart, muscle, brain, and other tissues. AGK is a 47,137-Da protein composed of 422 amino acids. It consists of a typical two-domain fold (DGK domain 1 and DGK domain 2) that mediates the phosphorylation of monoacylglycerols or diacylglycerols (Bektas et al., 2005; Qi et al., 2021). Also, AGK has an N-terminal α1 helix which is complete across the membrane, and a C-terminal key region bound to the membrane (Figure 2).

### Protein Kinase Function of AGK

Mitochondrial lipids maintain organelle homeostasis and participate in mitophagy and cytochrome C-mediated apoptosis (Mayr, 2015). AGK is originally found to be an important mitochondrial kinase involved in lipid metabolism. Its major kinase function is to catalyze the production of lysophosphatidic acid (LPA) and phosphatidic acid (PA) from monoacylglycerol (MAG) and diacylglycerol (DAG) (Bektas et al., 2005). AGK deficiency will lead to the disorder of mitochondria lipid membrane composition and affect mitochondrial homeostasis. In addition, it is believed that the ability of AGK to promote tumorigenesis involves the production of LPA, which is an effective signaling molecule that can drive cell growth and proliferation (Geraldo et al., 2021).

A decrease in the oxygen consumption rate and severe damage of mitochondrial ultrastructure were observed in AGK^–/–^ and AGK^G126E^ cells (a cell line in which the AGK enzyme activity is inhibited) (Vukotic et al., 2017), enlightening that effective mitochondrial respiration and mitochondrial structural integrity depend on AGK kinase activity. However, the specific mechanism remains to be clarified.

### Non-kinase Function of AGK

**FIGURE 3:**
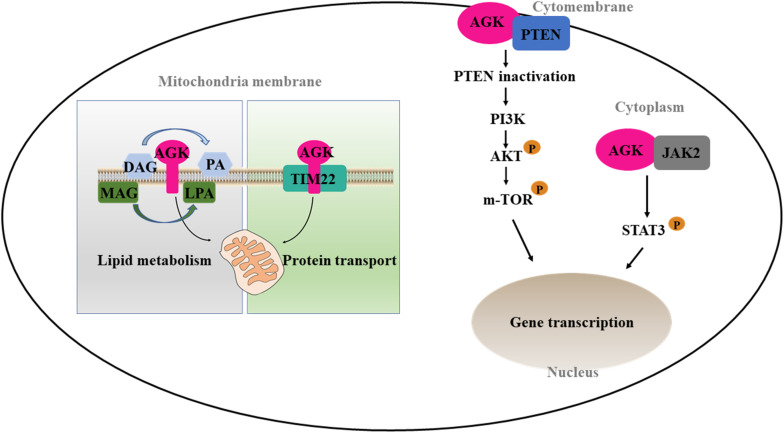
Kinase and non-kinase function of AGK. AGK as a kinase can phosphorylate MAG and DAG to form PLA and PA, respectively. As a non-kinase, AGK participates in mitochondrial protein transfer as a part of the TIM22 complex. Also, extramitochondrial AGK has a hand in the glycolysis of CD8 T cells and the formation of platelets by interacting with PTEN or JAK2, respectively. MAG, monoacylglycerol; DAG, diacylglycerol; LPA, lysophosphatidic acid; PA, phosphatidic acid; TIM22, translocase of the inner membrane 22; PTEN, phosphatase and tension homolog; PI3K, phosphatidylinositol 3-kinase; Akt, protein kinase B; mTOR, mammalian target of rapamycin; JAK2, Janus kinase 2; STAT3, signal transducer and activator of transcription 3.

On the one hand, AGK participates in phospholipid homeostasis as a mitochondrial kinase; on the other hand, AGK has a kinase-independent function (Figure 3). TIM22 complex mediates the insertion of multichannel transmembrane proteins into the mitochondrial membrane (Qi et al., 2021). Kang et al. (2018) discovered that AGK, as a subunit of the human TIM22 complex, plays an important role in stabilizing the TIM22 complex and regulating the import and assembly of mitochondrial carrier proteins [including adenine nucleotide transporter 1 (ANT1, also known as SLC25A4) and TDP-43 (Yu et al., 2020a)]. Surprisingly, the transport function of AGK does not depend on its kinase domain, which opens a new area for studying AGK as a non-kinase (Vukotic et al., 2017). Recently, Qi et al. (2021) revealed the Cryo-EM structure of the TIM22 complex, which includes TIM22, TIM9, TIM10a, TIM10b, TIM29, and AGK. This greatly enriched our understanding of the structure of the TIM22 complex.

CD8 T cells are important in adaptive immunity. Oxidative phosphorylation provides most of the energy for primitive and memory CD8 T cells (Vardhana et al., 2020). However, the increase of glycolysis is a prerequisite for the growth and activation of CD8 T cells (Varanasi et al., 2020). Loss of phosphatase and tension homolog (PTEN) in T cells leads to dysregulation of immune homeostasis (Lin et al., 2021). Moreover, PTEN is well known as a tumor inhibitor and phosphatidylinositol 3-kinase (PI3K)–mammalian target of rapamycin (mTOR) signaling inhibitor (Zheng et al., 2020). AGK on cytomembrane is involved in cell proliferation and activation by binding PTEN and activating the PI3K-mTOR pathway (Hu et al., 2019). On the contrary, AGK deficiency dramatically inhibits the antitumor function of CD8 T cells *in vivo* and *in vitro*, suggesting that targeting AGK in immune cells may be useful as an antitumor therapy.

Platelets are an important part of the blood, involved in hemostasis, tumor metastasis, and inflammation. The MPL/Janus kinase 2 (JAK2)/signal transducer and activator of transcription 3 (STAT3) signaling pathway is the most important pathway regulating megakaryocyte development and platelet formation (Plo et al., 2017). The most recent research declared that megakaryocyte/platelet-specific AGK-deficient mice show thrombocytopenia and splenomegaly. Cytoplasmic AGK promotes JAK2/STAT3 signal transduction by binding to JAK2 in megakaryocytes/platelets, ultimately inducing the formation of platelets (Jiang et al., 2020). These results suggest that AGK may be a new strategy for the treatment of thrombocytopenia and thrombocytopenia-related diseases.

Whether AGK kinase or kinase-independent functions are activated may depend on specific cell types and subcellular localization, and more wide-ranging studies are needed to explore the roles of AGK in specific organs and cell types.

### Regulation of AGK Activity

MicroRNA (miRNA) are known to play important roles in cancer progression by directly downregulating multiple targets (Goodall and Wickramasinghe, 2021). MiR-194 and miR-610 inhibit the proliferation and invasion of tumor cells by downregulating the expression of AGK (Chi, 2015; Yao et al., 2019), suggesting that AGK is an important downstream target of some miRNA mediated antitumor effects.

The Hippo signaling pathway is a newly discovered signal transduction pathway of tumors targeted for inhibition that is widely involved in cellular processes (Yu et al., 2015; Kulkarni et al., 2020). In gastric cancer, AGK is involved in tumor progression through the Hippo pathway. On the one hand, AGK, as the upstream matrix of Yes-associated protein 1 (YAP1), can induce the nuclear localization of YAP1 to enhance the transcriptional activity of YAP1/transcriptional enhanced associate domain (TEAD)s, and eventually lead to the proliferation, invasion, and epithelial–mesenchymal transition of cancer cells (Huang et al., 2020). On the other hand, the activity of AGK is regulated by the transcription of the large tumor suppressor (LATS)1/2 proteins upstream in the Hippo signaling pathway as their downstream target (Huang et al., 2020).

**FIGURE 4:**
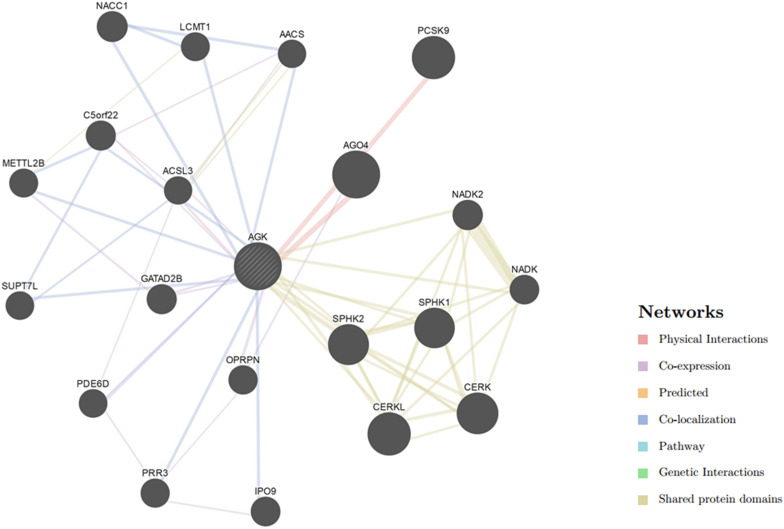
Gene interaction network of AGK. The gene–gene interaction network was generated by GeneMANIA (Warde-Farley et al., 2010). AGO4, argonaute 4; RISC, catalytic component; CERKL, ceramide kinase like; PCSK9, proprotein convertase subtilisin/kexin type 9; CERK, ceramide kinase; SPHK1, sphingosine kinase 1; SPHK2, sphingosine kinase 2; NACC1, nucleus accumbens associated 1; NADK2, NAD kinase 2, mitochondrial; GATAD2B, GATA zinc finger domain containing 2B; C5orf22, chromosome 5 open reading frame 22; IPO9, importin 9; NADK, NAD kinase; OPRPN, opiorphin prepropeptide; PRR3, proline rich 3; METTL2B, methyltransferase like 2B; LCMT1, leucine carboxyl methyltransferase 1; PDE6D, phosphodiesterase 6D; SUPT7L, SPT7-like STAGA complex gamma subunit; AACS, acetoacetyl-CoA synthetase; ACSL3, acyl-CoA synthetase long-chain family member 3.

**FIGURE 5:**
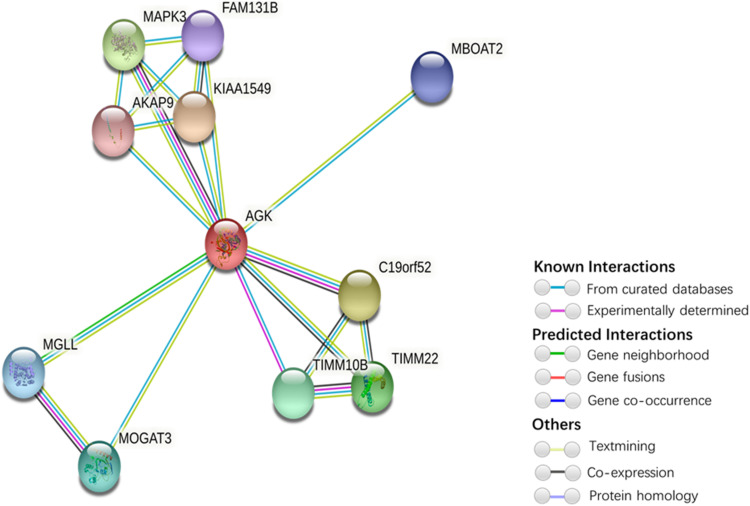
Protein interaction network of AGK-related proteins. The protein–protein interaction network was generated by STRING (Szklarczyk et al., 2019). Network nodes represent proteins. Edges represent protein–protein associations (associations are meant to be specific and meaningful, i.e., proteins jointly contribute to a shared function; this does not necessarily mean they are physically binding each other). Kiaa1545, UPF0606 protein KIAA1549; C19orf52, mitochondrial import inner membrane translocase subunit Tim29; MAPK3, mitogen-activated protein kinase 3; TIMM22, mitochondrial import inner membrane translocase subunit Tim22; TIMM10B, mitochondrial import inner membrane translocase subunit Tim10 B; MOGAT3, 2-acylglycerol O-acyltransferase 3; MGLL, monoglyceride lipase; MBOAT2, lysophospholipid acyltransferase 2; FAM131B, protein FAM131B; AKAP9, A-kinase anchor protein 9.

Our understanding of the regulation of AGK is currently based on very few studies (Table 1). The mechanism by which the upstream/downstream signals of AGK are regulated to initiate specific responses remains elusive. To gain a better understanding of the regulatory mechanisms of AGK, we analyzed and predicted the possible interaction gene (Figure 4) and protein (Figure 5) networks of AGK using GeneMANIA and STRING data to provide a reference for those who are interested in AGK research in the future.

**TABLE 1:** List of factors reported regulating AGK expression or activity.

Name	Cell types	Phenotypes	References
miR-194	Oral squamous cell carcinoma	Downregulation of AGK leads to antitumor	Chi, 2015
miR-610	Oral squamous cell carcinoma	Downregulation of AGK leads to antitumor	Yao et al., 2019
YAP1/TEADs	Gastric cancer	Upregulation of AGK leads to promote tumor	Huang et al., 2020

## AGK and Sengers Syndrome

Sengers syndrome is an autosomal recessive disorder caused by nuclear AGK gene mutation and was firstly described by Sengers et al. (1975). The clinical manifestation of Sengers syndrome closely resembles that of mitochondrial ATP synthesis disorder and cardiolipin metabolism deficiency. The predominant symptoms of the disease are congenital cataracts, hypertrophic cardiomyopathy, skeletal myopathy, exercise intolerance, and lactic acidosis (Mayr et al., 2012; Beck et al., 2018). Patients with severe cases die in infancy, while people with mild cases can survive for decades (Haghighi et al., 2014). Most patients died of heart failure caused by hypertrophic cardiomyopathy (Haghighi et al., 2014).

An increasing number of reports have investigated families with Sengers syndrome and *via* whole-exome sequencing identified different types of mutations in the AGK gene, and patients with different AGK mutations show clinical heterogeneity (Mayr et al., 2012; Siriwardena et al., 2013; Haghighi et al., 2014; Beck et al., 2018; Aggarwal et al., 2021). Adenine nucleotide transporter 1 (ANT1, also known as SLC25A4) is a mitochondrial protein with dual functions. It participates not only in metabolism through the regulation of ATP/ADP release in mitochondria but also in the regulation of cell death as a part of the mitochondrial permeability transition pore (Zhao et al., 2020b). In, Johnston et al. (2002) found that SLC25A4 activity decreased in two Sengers syndrome families without SLC25A4 gene mutation. Because of the importance of phospholipid metabolism for mitochondrial function, the main function of AGK is to produce LPA and PA through phosphorylation of MAG and DAG (Bektas et al., 2005). Mayr et al. (2012) speculated that AGK affects the stability of SLC25A4 by affecting the metabolism of mitochondrial phospholipids and suggested that Sengers syndrome is a mitochondrial disease associated with the lack of mitochondrial carrier SLC25A4. Importantly, SLC25A4 knockout mice exhibit phenotypes of hypertrophic cardiomyopathy, exercise intolerance, and lactic acidemia, which are extraordinarily similar to the symptom of Sengers syndrome. In addition, we were surprised to find that a newly published study drew the mitochondrial proteome map of fibroblasts from patients with Sengers syndrome by using the quantitative proteomics method and found that the enzymes related to mitochondrial carbon metabolism were downregulated, which was also detected in AGK-deficient cell lines (Jackson et al., 2021). These results suggest that the imbalance of carbon metabolism may be involved in the mitochondrial dysfunction associated with AGK deficiency, which provides a new insight for us to understand mitochondrial diseases, although AGK mutation is the root cause of Sengers syndrome. Although impaired mitochondrial protein input, the disorder of lipid metabolism, and one-carbon metabolism may contribute to the pathogenesis of Sengers syndrome, it is unfortunate that there is no method or drug available to cure this fatal disease. Until recently, some researchers have proposed that one-carbon metabolic disorder is a new molecular feature in the biology of Sengers syndrome, and serine supplementation may be a promising treatment for AGK deficiency (Jackson et al., 2021).

## AGK in Cancer

**FIGURE 6:**
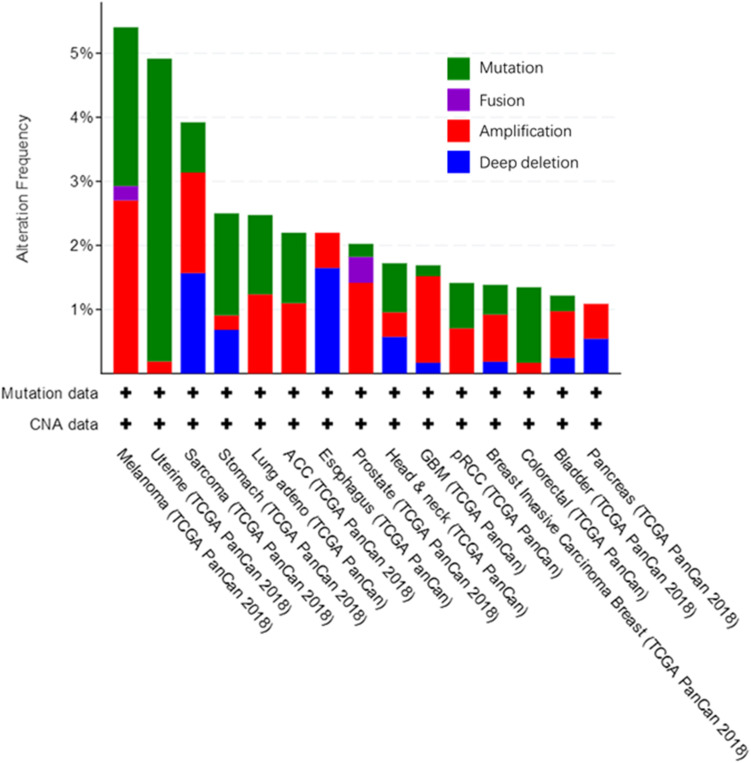
Alteration frequency in AGK gene across various cancers. All these data are concluded in cBioPortal, from TCGA Pan-Cancer Atlas Studies which cover 10,953 patients/10,967 samples.

**FIGURE 7:**
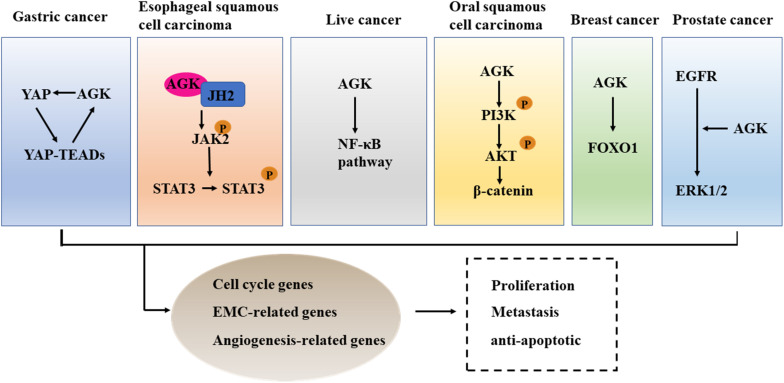
The cross talk between AGK and cancer-related pathways. AGK is participating in the differentiation, metastasis, and anti-apoptosis of tumor cells by adjusting Hippo-YAP1, JAK2/STAT3, NF-κB, and PI3K/Akt signaling pathways, FOXO1, and EGF transcription factor. YAP1, Yes-associated protein 1; TEAD, transcriptional enhanced associated domain; JH2, Janus kinase homology 2; JAK2, Janus kinase 2; STAT3, signal transducer and activator of transcription 3; NF-κB, nuclear factor-kappa B; PI3K, phosphatidylinositol 3-kinase; Akt, protein kinase B; FOXO1, forkhead box O1; EGFR, epidermal growth factor receptor; ERK, extracellular regulated protein kinases.

We used the cBioPortal tool (Cerami et al., 2012) to analyze the frequency of AGK mutation types in various malignant tumors (Figure 6) and found that AGK mutations are the most common, amplification and deletion mutations are found in some cancers, and fusions are found only in melanoma and prostate cancer. Moreover, the abnormal expression of AGK in various types of tumors has been reported (Table 2) and is involved in many processes of tumor biology, such as proliferation, migration, metastasis, and even drug resistance. Therefore, AGK, as an oncogene, may become a potential target for cancer treatment. Due to the heterogeneity and genetic instability of cancer, AGK seems to play a core role in cancer progression through different signaling pathways and molecules, such as the Hippo-YAP1, JAK2/STAT3, nuclear factor-kappa B (NF-κB), and PI3K/protein kinase B (Akt) signaling pathways as well as the forkhead box O1 (FOXO1) and epidermal growth factor (EGF) transcription factors (Figure 7). Next, we will introduce the specific mechanism of AGK involved in tumor progression from the perspective of the AGK-regulated signaling pathway.

**TABLE 2:** Expression of AGK in human tumor tissue compared with normal.

Cancer type	Expression	Analyzed level	Role/function	References
Gastric cancer	High	RNA/protein	Oncogenic	Huang et al., 2020
Nasopharyngeal carcinoma	High	RNA/protein	Oncogenic	Zhao et al., 2020a
Renal cell carcinoma	High	RNA/protein	Oncogenic	Zhu et al., 2020
Oral squamous cell carcinoma	High	RNA/protein	Oncogenic	Chi, 2015; Yao et al., 2019
Glioma	High	RNA/protein	Oncogenic	Liu et al., 2016
Cervical squamous cell cancer	High	RNA/protein	Oncogenic	Sun et al., 2016
Hepatocellular carcinoma	High	RNA/protein	Oncogenic	Cui et al., 2014
Breast cancer	High	Protein	Oncogenic	Wang et al., 2014
Esophageal squamous cell carcinoma	High	Protein	Oncogenic	Chen et al., 2013
Prostate cancer	High	RNA/protein	Oncogenic	Bektas et al., 2005; Spiegel and Milstien, 2005; Nouh et al., 2009

### Hippo-YAP1

The overexpression of YAP/transcriptional coactivator with PDZ-binding motif (TAZ), downstream effectors in the Hippo signaling pathway, leads to uncontrolled cell proliferation and malignant transformation (Dey et al., 2020). Therefore, an anomalously high expression of YAP/TAZ has been proved to be an independent predictor of tumor prognosis and an indicator of cell proliferation, metastasis, and poor survival (Kulkarni et al., 2020). In gastric cancer, YAP1/TAZ acts as an oncogene and promotes tumor formation by co-transcription with the TEAD transcription factor (Qiao et al., 2018). AGK was able to enhance the expression of nuclear YAP1 protein and then induce the transcriptional activity of the YAP1/TEAD gene (Huang et al., 2020). Therefore, targeting the expression or activity of AGK seems to be a therapeutic target to control the progression of gastric cancer.

### JAK/STAT

The JAK/STAT signaling pathway is constitutively activated in many kinds of cancer (Lin and Farooqi, 2020), and inhibition of its activation can suppress the occurrence and progression of cancer (Toh et al., 2020). In esophageal squamous cell carcinoma (ESCC), AGK directly binds to the Janus kinase homology 2 (JH2) domain of JAK2 to block the inhibition of JAK2 mediated by JH2, which leads to the activation of the JAK2/STAT3 signaling pathway and enhances the tumorigenicity of ESCC cells *in vivo* and *in vitro* (Chen et al., 2013). Additionally, AGK expression was positively correlated with STAT3 activation in lung cancer and breast cancer (Wang et al., 2014). Therefore, inhibiting the expression of AGK or using competitive AGK peptide to inhibit AGK-JAK interaction can be used as a novel and valuable method to block the activation of JAK/STAT3 in some tumors, such as ESCC (Chen et al., 2013).

### NF-κB

As a double-edged sword, the role of the NF-κB signal in cancer is complex (Yu et al., 2020b). On the one hand, NF-κB activation can promote the proliferation, invasion, and angiogenesis of tumor cells. On the other hand, the activation of atypical NF-κB can promote the apoptosis of cancer cells (Taniguchi and Karin, 2018). Studies have confirmed that NF-κB is abnormally activated in hepatocellular carcinoma and plays an important role in the malignant transformation of tumors (Czauderna et al., 2019). The overexpression of AGK promotes angiogenesis and enhances the resistance of tumor cells to apoptosis by activating the NF-κB signal in hepatocellular carcinoma (Cui et al., 2014). Therefore, targeted inhibition of AGK may be a favorable strategy for anti-angiogenesis and pro-apoptotic cancer therapy.

### PI3K/Akt

PI3K is a kind of lipid kinase that can transmit intracellular signal cascade and regulate a variety of cellular physiological processes (Koch et al., 2021). Protein kinase B (AKT) is an important downstream effector of PI3K signal transduction that regulates a variety of signaling pathways, including the inhibition of cell apoptosis, stimulation of cell growth, and regulation of cell metabolism (Hoxhaj and Manning, 2020). Activated PI3K/AKT can catalyze the phosphorylation of a series of proteins, ultimately promoting the growth and proliferation of tumor cells, inhibiting apoptosis, promoting invasion and metastasis, regulating the growth and angiogenesis of endothelial cells, and increasing the sensitivity to radiation (Lee et al., 2015; Alzahrani, 2019). MiRNAs are small non-coding RNAs that control the specificity of gene expression (McGeary et al., 2019). Increasing evidence shows that miRNAs regulate gene expression by targeting messenger RNAs (mRNAs) and thus participate in the process of malignant tumorigenesis (Goodall and Wickramasinghe, 2021). For example, in oral squamous cell carcinoma, miR-194 can inhibit the PI3K/Akt/FOXO3a signaling pathway by inhibiting AGK, resulting in the decreased expression of cyclin D1 and the increased expression of P21, which ultimately inhibits the proliferation and cell cycle progression of cancer cells (Chi, 2015). Additionally, a study of renal cell carcinoma and nasopharyngeal carcinoma found that AGK is involved in tumor progression and lymph node metastasis by activating the PI3K/Akt pathway (Zhu et al., 2020).

### FOXO1

FOXO1 is a member of the forkhead box containing an O subfamily of transcription factors (Deng et al., 2018). It plays an important role in many biological processes, including cell cycle arrest, cell death, apoptosis, stress response, cell differentiation, and metabolism (Kim et al., 2018). FOXO1 is downregulated in a variety of cancers and is considered to be a tumor suppressor (Jiang et al., 2018). The overexpression of AGK can reduce the activation of FOXO1 activity and its downstream targets, thereby enhancing the proliferation and tumorigenicity of cancer cells (Wang et al., 2014).

### EGF

The proliferation of many types of cancer cells is partly controlled by the autocrine EGF stimulation loop because epidermal growth factor receptor (EGFR) is consistently overexpressed in these cells (Sabbah et al., 2020). AGK in prostate cancer cells increases the formation and secretion of LPA, which leads to the transactivation of EGFR and the activation of the downstream MAPK signaling pathway, resulting in the growth of cancer cells. When the endogenous AGK was removed, the activation of extracellular regulated protein kinases (ERK)1/2 and cell proliferation induced by EGF was significantly inhibited (Spiegel and Milstien, 2005). Therefore, targeted inhibition of AGK may provide additional therapeutic benefits for patients with prostate cancer.

### Drug Resistance

EGFR mutant non-small cell lung cancers are resistant to EGFR tyrosine kinase inhibitor (EGFR-TKI) (Westover et al., 2018), and BRAF fusion is one of the reasons for their resistance (Yu et al., 2013). Lung cancer patients show secondary resistance to EGFR TKIs due to acquired AGK/BRAF fusion (Vojnic et al., 2019; Boyle et al., 2020), which suggests that AGK is involved in some acquired tumor resistance, but the specific AGK mechanism in this context needs to be clarified.

### Prognosis

Through the data analysis results of the relevant data of the protein atlas database (Table 3), we found that the high expression of AGK does not seem to predict the prognosis of tumor patients. While some studies have indicated that high AGK expression is associated with a poor prognosis for patients with certain types of cancer, AGK may be used to identify the risk of patients and guide personalized treatment. There seems to be a contradiction. However, we notice that the database takes the whole population as the research object for overall survival prognosis analysis, while the related research reports involve some specific groups and statistics of the overall survival rate, clinical stage, Fuhrman classification, recurrence with metastasis, and vital status, which may be the reason for the difference in statistical outcomes between the two. Since tumor prognosis is related to many factors, whether AGK can be used as a prognostic indicator of pan-cancer in the whole population remains to be supported by further clinical investigations and surveys.

**TABLE 3:** Analysis of cancer patient survival correlated with AGK expression.

Cancer type	Prognostic	*p*-value	% 5-year survival		Expression (n)		FPKM best cutoff		Median expression
			High	Low	High	Low		
Breast	No	0.11	84	80	391	684		5.32	4.88
Cervical	No	0.14	71	64	79	212		5.5	4.66
Colorectal	No	0.28	61	60	429	168		4.1	4.95
Endometrial	No	0.013	65	81	138	403		5.76	4.77
Glioma	No	0.0081	7	15	105	48		6.38	7.17
Head and neck	No	0.054	51	44	162	337		4.33	3.57
Liver	No	0.022	40	51	107	258		3.16	2.64
Lung	No	0.037	50	43	284	710		6.14	5.13
Melanoma	No	0.2	42*	41*	47	55		6.33	6.12
Ovarian	No	0.034	36	21	281	92		4.64	5.6
Pancreatic	No	0.11	31	18	131	45		3.08	3.63
Prostate	No	0.0062	94	99	99	395		5.67	4.83
Renal	No	0.079	69	68	198	679		6.74	5.06
Stomach	No	0.093	50	28	111	243		4.42	3.86
Testis	No	0.096	100	96	49	85		5.38	4.84
Thyroid	No	0.33	92	96	393	108		3.53	4.11
Urothelial	No	0.12	41	41	310	96		3.7	4.61

The data from Protein Atlas (https://www.proteinatlas.org/ENSG00000006530-AGK/pathology). FPKM, Fragments per Kilobase of exon per Million reads. *3-year survival.

## AGK-Related Mouse Models

To date, some investigators have tried to elucidate additional features of AGK by studying AGK-related genes in mice (Table 4). Agk^fl/fl^Pf4-Cre mice (megakaryocyte/platelet specific knockout mice) showed abnormal platelet development, similar to some Sengers syndrome patients with thrombocytopenia (Jiang et al., 2020). Additionally, Agk^fl/fl^Cd4-Cre mice, Agk^fl/fl^Cd4-Cre OT-I mice, and Agk^fl/fl^Cd4-Cre OT-II mice all showed weak antitumor ability (Hu et al., 2019). Agk^G126E/G126E^ mice and Agk^G126E/G126E^ OT-I mice, which were generated after G126E knock-in, showed uncontrolled tumor growth and lethality (Hu et al., 2019). However, the Agk^fl/fl^Pten^fl/fl^Cd4-Cre mice with PTEN knockout showed increased glycolysis in CD8 T cells, which led to the antitumor effect (Hu et al., 2019). AGK was necessary for the antitumor function of these CD8 T cells.

**TABLE 4:** The summary of AGK mouse models.

Mouse model	Functional characteristics	References
Agk^–/–^	Thrombocytopenia	Jiang et al., 2020
Agk^fl/fl^Pf4-Cre+	Megakaryocyte development impaired	Jiang et al., 2020
Agk^fl/fl^Cd4-Cre	Displayed a weaker ability to suppress tumor growth	Hu et al., 2019
Agk^fl/fl^Cd4-Cre OT-I		Hu et al., 2019
Agk^fl/fl^Cd4-Cre OT-II		Hu et al., 2019
Agk^fl/fl^Pten^fl/fl^Cd4-Cre	Increase glycolysis and cell growth	Hu et al., 2019
Agk^G126E/G126E^	Uncontrolled tumor growth and lethality	Hu et al., 2019
Agk^G126E/G126E^ OT-I		Hu et al., 2019

## Conclusion and Prospects

AGK, as a mitochondrial lipid kinase, has multiple kinases and kinase-independent biological functions, and its mutation leads to Sengers syndrome, which is characterized by multiple-organ dysfunction. As an effective oncogene, AGK is involved in the occurrence and development of a variety of cancers. Inhibiting its expression in certain cancer cells has led to anticancer effects, indicating that AGK is a potential therapeutic target in a variety of cancers. AGK shows diverse functions in specific cell and subcellular localization through different mechanisms, which helps us to develop antitumor strategies targeting AGK from different perspectives. By targeting the subcellular localization of AGK, or destroying its kinase or non-kinase function, it can be a promising antitumor strategy. However, there is no targeted and selective inhibitor for AGK, and one needs to be further developed and verified by drug researchers. The function of AGK in physiological and pathological conditions has not been fully elucidated. First, the diverse regulation of AGK in genomics, epigenetics, and posttranslational modification is rarely studied. Second, the function of AGK as a non-kinase in different cells is worthy of attention and exploration. In addition to tumor diseases, the roles of AGK in various systemic diseases remain unclear. In the future, it will be necessary to further study the interaction pathways and precise molecular targets of AGK to further clarify its physiological and pathological mechanisms.

## Author Contributions

BC, ZH, and XZ participated in designing and writing the manuscript. All authors contributed to the article and approved the submitted version.

## Conflict of Interest

The authors declare that the research was conducted in the absence of any commercial or financial relationships that could be construed as a potential conflict of interest.
